# A 12-Week Commercial Web-Based Weight-Loss Program for Overweight and Obese Adults: Randomized Controlled Trial Comparing Basic Versus Enhanced Features

**DOI:** 10.2196/jmir.1980

**Published:** 2012-04-25

**Authors:** Clare E Collins, Philip J Morgan, Penelope Jones, Kate Fletcher, Julia Martin, Elroy J Aguiar, Ashlee Lucas, Melinda J Neve, Robin Callister

**Affiliations:** ^1^Nutrition and DieteticsSchool of Health Sciences, Faculty of HealthThe University of NewcastleCallaghan, NSWAustralia; ^2^Priority Research Centre in Physical Activity and NutritionThe University of NewcastleCallaghan, NSWAustralia; ^3^School of EducationFaculty of Education & ArtsThe University of NewcastleCallaghan, NSWAustralia; ^4^SP Health Co. Pty LtdNorth SydneyAustralia; ^5^School of Biomedical Sciences and PharmacyFaculty of HealthThe University of NewcastleCallaghan, NSWAustralia

**Keywords:** Intervention, weight loss, Web-based intervention, randomized controlled trial, reducing diet, eHealth

## Abstract

**Background:**

The development and use of Web-based programs for weight loss is increasing rapidly, yet they have rarely been evaluated using randomized controlled trials (RCTs). Interestingly, most people who attempt weight loss use commercially available programs, yet it is very uncommon for commercial programs to be evaluated independently or rigorously.

**Objective:**

To compare the efficacy of a standard commercial Web-based weight-loss program (basic) versus an enhanced version of this Web program that provided additional personalized e-feedback and contact from the provider (enhanced) versus a wait-list control group (control) on weight outcomes in overweight and obese adults.

**Methods:**

This purely Web-based trial using a closed online user group was an assessor-blinded RCT with participants randomly allocated to the basic or enhanced 12-week Web-based program, based on social cognitive theory, or the control, with body mass index (BMI) as the primary outcome.

**Results:**

We enrolled 309 adults (129/309, 41.8% male, BMI mean 32.3, SD 4 kg/m^2^) with 84.1% (260/309) retention at 12 weeks. Intention-to-treat analysis showed that both intervention groups reduced their BMI compared with the controls (basic: –0.72, SD 1.1 kg/m^2^, enhanced: –1.0, SD 1.4, control: 0.15, SD 0.82; *P *< .001) and lost significant weight (basic: –2.1, SD 3.3 kg, enhanced: –3.0, SD 4.1, control: 0.4, SD 2.3; *P *< .001) with changes in waist circumference (basic: –2.0, SD 3.5 cm, enhanced: –3.2, SD 4.7, control: 0.5, SD 3.0; *P *< .001) and waist-to-height ratio (basic: –0.01, SD 0.02, enhanced: –0.02, SD 0.03, control: 0.0, SD 0.02; *P *< .001), but no differences were observed between the basic and enhanced groups. The addition of personalized e-feedback and contact provided limited additional benefits compared with the basic program.

**Conclusions:**

A commercial Web-based weight-loss program can be efficacious across a range of weight-related outcomes and lifestyle behaviors and achieve clinically important weight loss. Although the provision of additional personalized feedback did not facilitate greater weight loss after 12 weeks, the impact of superior participant retention on longer-term outcomes requires further study. Further research is required to determine the optimal mix of program features that lead to the biggest treatment impact over time.

**Trial Registration:**

Australian New Zealand Clinical Trials Registry (ANZCTR): 12610000197033; http://www.anzctr.org.au/trial_view.aspx?id=335159 (Archived by WebCite at http://www.webcitation.org/66Wq0Yb7U)

## Introduction

The prevalence of overweight and obesity among adults is increasing worldwide [1]. Therefore, effective treatment programs with a large reach are required. Web-based weight-loss programs have emerged in response to the exponential growth in Internet access, as well as increasing use of the Internet as part of daily life and improved accessibility. In the United States, 66% of households have access to broadband Internet [2], while 72% of Australian households have access to the Internet [3]. Furthermore, many adults (61% in the United States) use the Internet to access information about health, nutrition, physical activity, and weight loss [4]. The multimedia capabilities of the Internet allow up-to-date, interactive, and individualized lifestyle programs to be provided, which endeavor to emulate traditional face-to-face consultations [5]. These programs also overcome several barriers of attending face-to-face consultations, such as poor accessibility [6], lack of anonymity [7], and participant burden associated with attendance.

However, Web-based weight-loss programs are an underevaluated treatment medium. A recent systematic review and meta-analysis examined the efficacy of 12 randomized controlled trials (RCTs) of Web-based weight-loss programs [8]. The results suggest that, in general, participants in these programs achieve similar weight-loss outcomes to control or minimal intervention groups. In addition, it has been suggested that Web-based programs with enhanced features (eg, tailored information and counseling) achieve greater weight loss than those that focus on information alone, although these studies are highly heterogeneous [8]. Further studies are required, as it has not yet been possible to establish the overall efficacy of Web-based interventions or the superiority of those with more enhanced features, due to the heterogeneity of study designs and therefore the small number of comparable studies.

Of the small number of Web-based programs that have been evaluated using an RCT, remarkably few are available to the public. Commercial Web-based weight-loss programs are likely to be the most accessible to consumers [9] but have rarely been independently evaluated [10]. Of the two RCTs of eDiets, a commercial Web-based weight-loss program in the United States, one found that after 12 months those using eDiets lost significantly less weight than those following a self-help manual (–1.1% vs –4.0%) [11], while the second compared eDiets with a structured behavioral Web-based program [12] and found the behavioral program achieved significantly greater weight loss after 12 months (–2.8% vs –5.5%). Overall, limited evidence exists for the efficacy of commercial Web-based programs as a viable obesity treatment option. Therefore, examining the efficacy of commercially available Web-based weight-loss programs on weight-related outcomes is warranted to increase the treatment options for people seeking to engage in commercial treatment programs, especially those who have limited options in their region.

The primary aim of this study was to determine whether there was a significant difference in body mass index (BMI) posttreatment among participants randomly assigned to a standard (basic) 12-week commercial Web-based weight-loss program versus a version of this program with additional online features and personalized e-feedback and reminder calls (enhanced) versus a wait-list control (control). We hypothesized that the reduction in BMI would be greater in the basic and enhanced groups than in the control group, with the BMI reduction greater in the enhanced than in the basic group.

## Methods

The present study was an assessor-blinded RCT with a 12-week follow-up. The methods have been published in detail [13]. Briefly, overweight and obese adults were recruited offline and enrolled by research assistants at the University of Newcastle from October to December 2009 from the Hunter community in New South Wales, Australia. Eligibility criteria were age 18 to 60 years, BMI 25 to 40 kg/m^2^, not participating in other weight-loss programs, passing a health screen [14], being available for in-person assessments, and having access to a computer with email and Internet services, although neither computer nor eHealth literacy was assessed. Exclusion criteria were pregnancy or trying to conceive, major medical problems, orthopedic problems, recent weight loss of ≥4.5 kg, and medications affecting or affected by weight loss. Trial sample size calculations have been previously published [13].

### Stratification and Randomization

**Figure 1:**
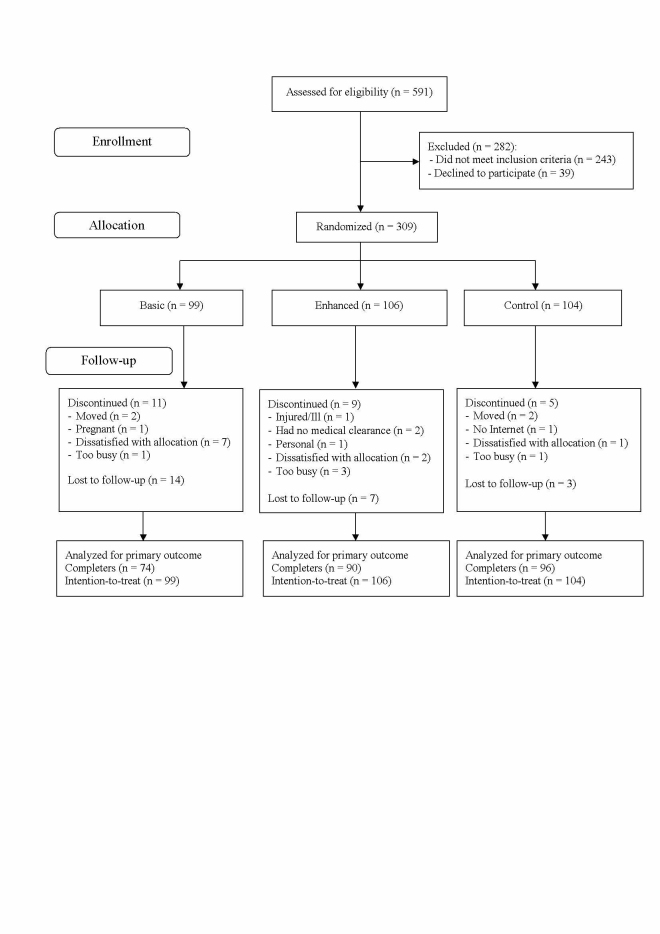
Flow of participants through the three groups (control, basic, enhanced) of a web-based weight-loss randomized controlled trial.

Once written consent was obtained and baseline assessments completed, participants were stratified by sex and BMI category (25 to <30; ≥30 to <35 or ≥35 to 40 kg/m^2^). They were randomly allocated to one of the three groups between October and December 2009 (Figure 1) using a stratified randomized block design with variable blocks length (either 3 or 6) generated by the statistician. A researcher not involved in data collection distributed sequentially numbered sealed envelopes with allocation details and a login code. Participants were given an instruction sheet and the Web address and asked to set up their own login. We also gave them a toll-free number to call if they experienced any difficulties in logging in. No training on program use was provided to participants in order to mirror the program engagement that commercial users would experience and to increase external validity. The groups were (1) control: a wait-list control group who were not provided with access to the weight-loss program website and were asked to refrain from participating in other weight-loss programs for 12 weeks, (2) basic: who were provided with free access to the basic (standard) Web-based program that was commercially available at that time and did not change, and (3) enhanced: who were provided with free access to an enhanced version of the Web-based program that was provided in a closed test environment. Both basic and enhanced group participants were advised to use the online diary a minimum of 4 times per week to record their dietary intake and physical activity, and to enter a weekly weight. Participants were blinded to allocation for the basic and enhanced groups, but not to the control. Research assistants were blinded to allocation for all groups. At each time point all were reminded to not discuss group allocation at assessments.

#### Web-Based Weight-Loss Programs

The Web-based program was underpinned by social cognitive theory [15] and targeted key mediators of behavior change, including self-efficacy, goal setting, and self-monitoring of weight, body measurements, exercise, and diet; outcome expectations (knowledge-based Web components); modeling (interactive website features and demonstrations); and social support (forums, blogs, feedback, email, and telephone contact). The interventions were Web based and delivered for 12 weeks, with new program content provided weekly by SP Health Co Pty Ltd, a commercial Web-based weight-loss program provider in Australia, under the name The Biggest Loser Club. Participation was in a quasi-anonymous manner. The reach [16], retention [17], and weight loss achieved by real-world participants [18] in the basic program have been previously evaluated.

The basic program had the following features: individualized daily calorie targets to facilitate 0.5–1 kg of weight loss per week; goal-setting options; Web-based food and exercise diary; weekly calorie-controlled, low-fat menu plans and grocery list; weekly physical activity plan based on exercise preferences; weekly educational tips and challenges; Web-based community forums; daily and weekly calculations of energy balance and nutrition summary compared with recommended nutrient targets; weekly email newsletters with alerts to new relevant content; self-monitoring of weight, and waist and hip girths; graphical display of changes in body measurement data and body (BMI) silhouette; and automated weekly reminders to enter weight. The enhanced program included access to the Web-based program described above plus (1) personalized, system-generated enrollment reports that suggested appropriate weight-loss goals and key behavior changes required for success, (2) weekly automated, system-generated, personalized e-feedback for key elements of diet and physical activity based on diary entries, usage patterns for website features, and level of success with weight loss, and (3) an escalating reminder schedule to use the diary, visit the program site, and enter a weekly weight, which was as an initial reminder email, then a short message service text message if they did not respond, then a courtesy reminder phone call if a weekly weight was still not entered.

Participants accessed the website using their usual Internet connection, at any time of the day or night that suited them.

#### Outcome Measures

Participant assessments were conducted at baseline and 12 weeks in the Human Performance Laboratory at the University of Newcastle, Callaghan campus [13]. Assessors of the main outcome measures, including those performing anthropometric and blood pressure measurements and blood collection, were blinded to participant group allocation at baseline and 12 weeks. We asked participants not to inform assessors of their group allocation.

The primary outcome, BMI, was calculated as weight (in kilograms) divided by height (in meters) squared. Height was measured to 0.1 cm using the stretch stature method on a Harpenden portable stadiometer (Holtain Limited, Croswell, Crymych, Pembrokeshire, UK). Weight was measured in light clothing, without shoes, on a digital scale to 0.01 kg (CH-150kp; A&D Mercury Pty Ltd, Adelaide, Australia).

Waist circumference was measured to 0.1 cm using a nonextensible steel tape (KDSF10-02; KDS Corporation, Osaka, Japan) at two points: (1) level with the umbilicus, and (2) at the narrowest point between the lower costal border and the umbilicus. Systolic and diastolic blood pressure and heart rate were measured using an automated blood pressure monitor (NISSEI/DS-105E digital electronic blood pressure monitor; Nihon Seimitsu Sokki Co Ltd, Gunma, Japan) under standardized conditions.

Blood samples were collected after an overnight fast and analyzed for lipids (total cholesterol, low-density lipoprotein and high-density lipoprotein cholesterol, and triglycerides), glucose, and insulin using standard automated techniques at a single National Association of Testing Authorities-accredited pathology service.

Dietary intake was assessed using the Australian Child and Adolescent Eating Survey, a 135-item semiquantitative food frequency questionnaire used previously in Australian youth [19] and currently being validated in adults. We asked participants to self-report frequency of consumption over the previous 6 months using the paper-based tool at baseline and over the previous 12 weeks at the follow-up assessment. Frequency options ranged from never up to ≥4 times per day. Completed food frequency questionnaires were scanned and nutrient intakes computed using FoodWorks (version 3.02.581; Xyris Software, Highgate Hill, Australia) using the Australian AusNut 1999 database (All Foods) revision 14 and AusFoods (Brands) revision 5 (Food Standards Australia New Zealand, Canberra, Australia) to generate individual mean daily nutrient intakes. We used the paper-based Three-Factor Eating Questionnaire-R18 (TFEQ-R18) to measure cognitive restraint, uncontrolled eating, and emotional eating [20]. Quality of life was assessed using the paper-based SF-36, version 2.0 (QualityMetric Incorporated, Lincoln, RI, USA), a multipurpose, generic, short-form health survey consisting of an 8-scale profile of functional health and well-being scores and psychometrically based physical and mental health summary measures [21].

We used the International Physical Activity Questionnaire (short form) paper-based questionnaire to estimate total metabolic equivalents (METs) in minutes/week [22]. Pedometers were used to objectively measure steps per day for 7 consecutive days (Yamax Digi-Walker SW-700; Yamasa Tokei Keiki Co Ltd, Tokyo, Japan). The step counts were adjusted for additional physical activity self-reported by participants when the pedometers were not worn (eg, contact sports and swimming) or problematic (eg, cycling). We added 1000 steps for every 10 minutes of moderate activity and 2000 steps for every 10 minutes of vigorous activity.

### Ethics

The procedures followed were in accordance with the ethics standards of the University of Newcastle Human Ethics Research Committee. We obtained written informed consent from all participants. Institutional affiliations were displayed on paper-based information and consent forms, but not on the Web-based program materials.

### Statistical Analysis

Analysis of covariance was used to test for differences in weight loss between groups at 3 months after adjusting for baseline values. Analysis was on an intention-to-treat basis with participants who did not use the application included in the analysis, while baseline observation carried forward was used those lost to follow-up. The model was fitted using linear regression with BMI at 12weeks as the outcome variable, treatment group as the predictor variable of interest, and BMI at baseline included as a covariate. We based statistical significance of the primary efficacy analysis on Hochberg multiple testing procedures with the familywise error rate held at 2.5% because there will be an additional analysis at 18 months. All secondary hypothesis tests were performed using a 2-sided 5% significance level.

## Results

### Baseline Characteristics

Of the 591 people assessed for eligibility, 309 (129 men) were randomly allocated into the trial (Figure 1). Baseline characteristics did not differ between groups for any variable, other than general health scale (Table 1). Most of the participants were overweight (108, 35.0%) or in obese category 1 (122, 39.5%), most (280, 90.6%) were Australian born, and few (33, 11%) had ever smoked.

**Table 1:** Baseline characteristics of participants in a 12-week Web-based weight-loss (WL) program by intervention group.

Characteristic		Treatment group				*P *value^a^
		Control (n = 104)	Basic WL (n = 99)	Enhanced WL (n = 106)	Total (N = 309)	
**Sex, n(%)**						
	Men	44 (42%)	41 (41%)	44 (42%)	129 (42%)	.99
	Women	60 (58%)	58 (59%)	62 (58%)	180 (58%)	
**BMI group strata (kg/m^2^), n (%)**						
	25 to <30	36 (35%)	34 (34%)	38 (36%)	108 (35.0%)	.99
	30 to <35	42 (40%)	39 (39%)	41 (39%)	122 (39.5%)	
	35 to <40	26 (25%)	26 (26%)	27 (25%)	79 (26%)	
**Current or previous smoker, n (%)**						
	Never smoker	93 (91%)	85 (87%)	95 (90%)	273 (89%)	.59
	Current/former smoker	9 (9%)	13 (13%)	11 (10%)	33 (11%)	
**Highest level of education, n (%)**						
	School	27 (26%)	32 (32%)	31 (29%)	90 (29%)	.78
	Trade/diploma	37 (36%)	31 (31%)	43 (41%)	111 (36%)	
	University degree	26 (25%)	23 (23%)	19 (18%)	68 (22%)	
	Higher university degree	13 (13%)	13 (13%)	13 (12%)	39 (13%)	
**Weekly household income (A $), n (%)**						
	<$700	10 (10%)	9 (10%)	6 (6%)	25 (8.6%)	.72
	$700 to <$1000	6 (6%)	3 (3%)	7 (7%)	16 (6%)	
	$1000 to <$1400	12 (12%)	15 (16%)	9 (9%)	36 (12%)	
	$1500 or more	68 (69%)	62 (67%)	75 (75%)	205 (70%)	
	Don’t know/no answer	3 (3%)	4 (4%)	3 (3%)	10 (3%)	
**Country of birth, n (%)**						
	Australia	92 (89%)	90 (91%)	98 (92%)	280 (90.6%)	.73
	Other	11 (11%)	9 (9%)	8 (8%)	28 (9%)	
Age (years), mean (SD)		41.7 (9.4)	42.0 (10.9)	42.2 (10.2)	42.0 (102)	.94
**Physical measurements, mean (SD)**						
	Height (cm)	1.7 (0.1)	1.7 (0.1)	1.7 (0.1)	1.7 (0.1)	.65
	Weight (kg)	93.6 (13.9)	94.9 (15.4)	93.4 (14.6)	94.0 (14.6)	.75
	Body mass index (kg/m^2^)	32.2 (3.9)	32.3 (3.6)	32.3 (4.3)	32.3 (4.0)	.98
	Waist circumference (umbilicus) (cm)	107.2 (10.4)	106.9 (9.8)	106.6 (12.5)	106.9 (10.9)	.92
	Waist circumference (narrowest) (cm)	98.2 (11.4)	98.6 (11.5)	97.7 (11.7)	98.2 (11.5)	.86
	Waist (umbilicus) to height ratio	0.63 (0.07)	0.63 (0.06)	0.63 (0.08)	0.63 (0.07)	.91
	Waist (narrowest) to height ratio	0.58 (0.06)	0.58 (0.06)	0.57 (0.06)	0.58 (0.06)	.98
**Physiological measurements, mean (SD)**						
	Systolic blood pressure (mmHg)	122 (16)	121 (13)	121 (11)	122 (13)	.81
	Diastolic blood pressure (mmHg)	79 (10)	80 (11)	79 (10)	79 (10)	.75
	Pulse rate (beats/minute)	70 (10)	68 (9)	68 (10)	69 (10)	.55

**Blood tests, mean (SD)**						
	Total serum cholesterol (mmol/L)	5.1 (0.8)	5.2 (1.0)	5.0 (1.1)	5.1 (1.0)	.64
	LDL^b ^cholesterol (mmol/L)	3.0 (0.6)	3.1 (0.8)	3.0 (0.9)	3.0 (0.8)	.38
	HDL^c ^cholesterol (mmol/L)	1.3 (0.3)	1.3 (0.3)	1.3 (0.3)	1.3 (0.3)	.85
	Triglycerides (mmol/L)	1.8 (1.9)	1.6 (0.8)	1.8 (1.2)	1.7 (1.4)	.54
	LDL to HDL ratio	2.40 (0.79)	2.54 (0.80)	2.38 (0.79)	2.44 (0.79)	.35
	Glucose (mmol/L)	5.0 (1.4)	4.8 (0.6)	4.8 (0.6)	4.8 (0.9)	.20
	Insulin (mIU/L)	10.5 (7.5)	11.8 (13.1)	11.2 (13.1)	11.1 (11.5)	.73
**SF-36 scores, mean (SD)**						
	Physical functioning	85.4 (15.2)	86.1 (14.2)	82.5 (19.8)	84.6 (16.7)	.27
	Role physical	87.1 (19.9)	88.8 (16.8)	86.3 (19.5)	87.4 (18.8)	.63
	Bodily pain	60.9 (28.4)	61.4 (27.3)	61.2 (29.8)	61.2 (28.4)	.99
	General health	63.9 (18.5)	69.8 (16.8)	72.8 (18.8)	68.7 (18.5)	.004
	Vitality	78.1 (70.6)	69.3 (14.8)	81.4 (45.3)	76.8 (51.4)	.32
	Social functioning	81.4 (23.8)	84.5 (20.6)	85.0 (21.7)	83.5 (22.2)	.51
	Role emotional	86.5 (17.4)	89.9 (21.7)	89.4 (15.9)	88.5 (18.2)	.41
	Mental health	74.0 (17.3)	78.9 (16.5)	79.2 (14.8)	77.2 (16.4)	.06
**Physical activity, mean (SD)**						
	Total (MET^d ^minutes/week)	2724 (2732)	3024 (3029)	2846 (3127)	2863 (2964)	.80
	Step count/day	7971 (3511)	8664 (3773)	8680 (3752)	8427 (3677)	.34
**Three-Factor Eating Questionnaire-R18 scores, mean (SD)**						
	Cognitive restraint scale	13.0 (3.0)	13.3 (2.9)	13.1 (2.8)	13.2 (2.9)	.70
	Uncontrolled eating scale	20.9 (5.1)	20.9 (4.7)	20.8 (4.8)	20.9 (4.8)	.97
	Emotional eating scale	7.7 (2.5)	7.6 (2.2)	7.8 (2.6)	7.7 (2.4)	.86
Total energy intake (kJ/day), mean (SD)		10,311 (3229)	9958 (3223)	10,250 (3257)	10,175 (3229)	.71

**^a^** *P *values are from analysis of variance for continuous measures and from a chi-square test for categorical measures.

^b ^Low-density lipoprotein.

^c ^High-density lipoprotein.

^d ^Metabolic equivalent.

### Retention at 12 Weeks

The percentage of participants who attended the 12-week follow-up assessment was significantly different between treatment groups (*P *= .003). Participants randomly assigned to the basic group (74/99, 75%) were less likely (*P *= .001) to attend the 12-week visit than those in the control group (96/104, 92%), with the percentage attending from the enhanced group (90/106, 85%) not significantly different from either the control (*P *= .09) or basic condition (*P *= .07) (Figure 1).

### Changes in Weight, BMI, and Waist Circumference

Participants in the basic and enhanced groups lost significant amounts of weight whether expressed as BMI or kilograms lost (Table 2, Table 3), with the increase in weight in controls not statistically significant. Consequently, those randomly assigned to the basic and enhanced groups had statistically significant reductions in the primary outcome, BMI (kg/m^2^), compared with those in the control group. Waist circumferences decreased significantly more in the basic and enhanced groups than in the control group, and waist circumference measured at the narrowest point decreased significantly more in the enhanced group than in the basic group (Table 2, Table 3). Waist-to-height ratios decreased in the basic and enhanced groups compared with the control group.

### Secondary Outcomes

After 12 weeks we observed a statistically significant improvement in total serum cholesterol and systolic and diastolic blood pressures in those randomly assigned to the enhanced condition compared with control, with a nonsignificant benefit in those in the basic condition. There were no differences between groups in changes in any of the other plasma variables, including triglycerides, glucose, and insulin. There was a trend toward a greater reduction in pulse rate in the enhanced compared with control group (*P *= .06). There was no significant change in total physical activity METs (minutes/week), with the average step count per day decreasing in the controls but increasing in the basic and enhanced groups, and a significantly greater increase in enhanced relative to control (*P *= .005). While all groups decreased their energy intake (kJ/day), those in the enhanced group decreased theirs more than those in the control group (*P *= .03). There was no change in most of the subscales of the SF-36 quality-of-life questionnaire, with the exception of the general health scale, which improved more in the enhanced than in the control group (*P *= .03). Within the TFEQ-R18, the scales of cognitive constraint and uncontrolled eating also improved. Both basic and enhanced participants increased eating restraint and reduced uncontrolled eating compared with controls (*P *< .001).

**Table 2:** Mean (SD) change in variables from baseline to 12 weeks in each treatment group.

Variable	Total n	Treatment group		
		Control	Basic	Enhanced
Weight (kg)	309	0.36 (2.33)	–2.14 (3.32)	–2.98 (4.05)
Percentage weight loss (%)	309	0.44 (2.44)	–2.29 (3.51)	–3.26 (4.31)
Body mass index (kg/m^2^)	309	0.15 (0.82)	–0.72 (1.07)	–0.98 (1.38)
Waist circumference at umbilicus (cm)	309	0.26 (3.10)	–2.63 (3.99)	–3.18 (5.00)
Waist circumference at narrowest point (cm)	309	0.46 (3.02)	–1.96 (3.47)	–3.17 (4.69)
Waist to height ratio at umbilicus	309	0.00 (0.02)	–0.02 (0.02)	–0.02 (0.03)
Waist to height ratio at narrowest point	309	0.00 (0.02)	–0.01 (0.02)	–0.02 (0.03)
Systolic blood pressure (mmHg)	308	–1.09 (10.90)	–3.56 (9.35)	–4.95 (10.08)
Diastolic blood pressure (mmHg)	308	–0.35 (7.04)	–2.09 (7.74)	–3.02 (8.57)
Pulse rate (beats/minute)	306	–0.86 (6.54)	–0.99 (6.47)	–2.52 (6.28)
Total serum cholesterol (mmol/L)	309	0.08 (0.55)	–0.05 (0.51)	–0.17 (0.56)
LDL^a ^cholesterol (mmol/L)	271	0.09 (0.49)	0.03 (0.40)	–0.05 (0.43)
HDL^b ^cholesterol (mmol/L)	309	–0.00 (0.13)	0.01 (0.15)	–0.01 (0.16)
Triglycerides (mmol/L)	309	–0.22 (1.50)	–0.17 (0.58)	–0.23 (0.67)
LDL to HDL ratio	271	0.06 (0.40)	0.00 (0.36)	–0.03 (0.34)
Glucose (mmol/L)	307	–0.45 (0.91)	–0.35 (0.53)	–0.33 (0.55)
Insulin (mIU/L)	309	–0.76 (5.11)	–1.53 (12.76)	–1.55 (6.04)
Physical functioning (SF-36)	301	0.45 (14.27)	1.79 (22.64)	4.86 (17.22)
Role physical (SF-36)	301	1.92 (21.74)	2.04 (21.74)	3.07 (16.39)
Bodily pain (SF-36)	300	–2.08 (27.65)	0.20 (25.40)	0.85 (34.34)
General health (SF-36)	303	3.02 (11.90)	3.72 (12.17)	6.75 (12.42)
Vitality (SF-36)	297	14.11 (68.04)	2.36 (22.48)	12.91 (59.23)
Social functioning (SF-36)	298	1.36 (24.42)	0.26 (14.47)	4.48 (21.77)
Role emotional (SF-36)	303	1.32 (20.51)	2.47 (13.55)	3.07 (16.48)
Mental health (SF-36)	299	2.28 (15.66)	2.55 (15.68)	4.86 (13.26)
Total physical activity MET^c ^(minutes/week)	274	341.8 (3116)	151.4 (1946)	491.6 (2601)
Average step count per day	263	–61 (2480)	153 (2095)	867 (2947)
Cognitive restraint scale (TFEQ-R18)^d^	296	0.28 (2.50)	1.16 (2.48)	1.78 (3.34)
Uncontrolled eating scale (TFEQ-R18)	302	0.05 (3.03)	–1.58 (3.53)	–1.81 (3.74)
Emotional eating score (TFEQ-R18)	304	–0.32 (1.37)	–0.47 (1.38)	–0.63 (1.64)
Total energy intake (kJ/day)	304	–734 (2129)	–1003 (2498)	–1465 (2470)

^a ^Low-density lipoprotein.

^b ^High-density lipoprotein.

^c ^Metabolic equivalent.

^d ^Three-Factor Eating Questionnaire-R18.

**Table 3:** Absolute and least square mean (LSM) differences between groups at 12 weeks (intention-to-treat population).

Variable	Total n	Treatment group LSM (95% CI^a^)						Group effect (*P *value)
		Basic vs control		Enhanced vs control		Enhanced vs basic		
		Difference	95% CI	Difference	95% CI	Difference	95% CI	
Weight (kg)	309	2.48	1.38 to 3.58	3.34	2.26 to 4.42	0.86	–0.23 to 1.95	<.001
Percentage weight loss (%)	309	2.73	1.57 to 3.89	3.70	2.55 to 4.84	0.97	–0.19 to 2.12	<.001
Body mass index (kg/m^2^)	309	0.87	0.51 to 1.24	1.13	0.77 to 1.50	0.26	–0.11 to 0.63	<.001
Waist circumference at umbilicus (cm)	309	2.90	1.54 to 4.26	3.45	2.11 to 4.79	0.55	–0.81 to 1.91	<.001
Waist circumference at narrowest point (cm)	309	2.40	1.16 to 3.64	3.65	2.43 to 4.87	1.25	0.02 to 2.49	<.001
Waist to height ratio at umbilicus	309	0.02	0.01 to 0.03	0.02	0.01 to 0.03	0.00	–0.01 to 0.01	<.001
Waist to height ratio at narrowest point	309	0.01	0.01 to 0.02	0.02	0.01 to 0.03	0.01	–0.00 to 0.01	<.001
Systolic blood pressure (mmHg)	308	2.82	–0.17 to 5.81	4.24	1.31 to 7.17	1.42	–1.55 to 4.39	.003
Diastolic blood pressure (mmHg)	308	1.41	–0.96 to 3.78	2.54	0.22 to 4.87	1.13	–1.23 to 3.49	.04
Pulse rate (beats/minute)	306	0.31	–1.77 to 2.39	1.92	–0.12 to 3.95	1.60	–0.47 to 3.67	.06
Total serum cholesterol (mmol/L)	309	0.11	–0.06 to 0.28	0.25	0.08 to 0.42	0.14	–0.03 to 0.31	.003
LDL^b ^cholesterol (mmol/L)	271	0.04	–0.11 to 0.20	0.14	–0.01 to 0.29	0.10	–0.06 to 0.25	.09
HDL^c ^cholesterol (mmol/L)	309	0.01	–0.04 to 0.05	0.01	–0.03 to 0.06	0.02	–0.03 to 0.07	.59
Triglycerides (mmol/L)	309	0.07	–0.15 to 0.28	0.03	–0.18 to 0.25	0.03	–0.18 to 0.25	.78
LDL to HDL ratio	271	0.05	–0.08 to 0.18	0.09	–0.03 to 0.22	0.05	–0.08 to 0.17	.23
Glucose (mmol/L)	307	0.00	–0.17 to 0.17	0.03	–0.14 to 0.20	0.03	–0.14 to 0.21	.86
Insulin (mIU/L)	309	0.21	–2.07 to 2.50	0.46	–1.78 to 2.71	0.25	–2.02 to 2.53	.89
Physical functioning (SF-36)	301	1.78	–3.51 to 7.06	2.65	–2.54 to 7.83	0.87	–4.37 to 6.11	.47
Role physical (SF-36)	301	0.72	–4.83 to 6.26	0.05	–5.38 to 5.48	0.77	–4.72 to 6.26	.94
Bodily pain (SF-36)	300	2.91	–5.63 to 11.45	2.69	–5.66 to 11.04	0.22	–8.22 to 8.66	.67
General health (SF-36)	303	1.25	–2.63 to 5.13	4.24	0.45 to 8.04	2.99	–0.84 to 6.82	.03
Vitality (SF-36)	297	10.38	–5.56 to 26.32	1.12	–14.5 to 16.71	9.26	–6.49 to 25.01	.24
Social functioning (SF-36)	298	0.15	–6.07 to 6.36	2.05	–4.02 to 8.13	1.90	–4.26 to 8.07	.68
Role emotional (SF-36)	303	2.74	–2.02 to 7.51	1.37	–3.29 to 6.02	1.38	–3.34 to 6.10	.40
Mental health (SF-36)	299	1.02	–3.56 to 5.60	2.30	–2.18 to 6.78	1.28	–3.25 to 5.82	.48
Total physical activity MET^d ^(minutes/week)	274	96.51	–776 to 969.4	190.3	–661 to 1042	286.8	–568 to 1141	.72
Average step count per day	263	436	–485 to 1357	1225	339.3 to 2111	789	–130 to 1707	.005
Cognitive restraint scale (TFEQ-R18)^e^	296	1.05	0.16 to 1.93	1.57	0.72 to 2.43	0.52	–0.36 to 1.40	<.001
Uncontrolled eating scale (TFEQ-R18)	302	1.65	0.55 to 2.76	1.89	0.82 to 2.96	0.23	–0.86 to 1.33	<.001
Emotional eating score (TFEQ-R18)	304	0.18	–0.30 to 0.65	0.30	–0.16 to 0.77	0.13	–0.35 to 0.60	.30
Total energy intake (kJ/day)	304	397	–291 to 1084	782	100 to 1463	385	–304 to 1074	.03

^a ^Confidence interval.

^b ^Low-density lipoprotein.

^c ^High-density lipoprotein.

^d ^Metabolic equivalent.

^e ^Three-Factor Eating Questionnaire-R18.

There were significant between-group differences in the proportions of participants who lost 5 to <10% (control: 3%, basic: 18%, enhanced: 17%) or ≥10% of their baseline weight (control: 0%, basic: 18%, enhanced: 28%), or gained weight (control: 53%, basic: 22%, enhanced: 17%) (all *P *< .001).

## Discussion

The primary aim of this study was to determine whether using a commercial Web-based weight-loss program, with or without enhanced online features including personalized e-feedback and reminder calls, would lead to a greater reduction in BMI compared with each other or with a wait-list control group. This study demonstrated that participation in either version of the 12-week commercial Web-based weight-loss program (standard or enhanced with feedback) resulted in statistically significant and clinically important objectively measured weight loss. Many desirable improvements in secondary risk markers for chronic disease were achieved, irrespective of the program version used. This suggests that the fundamental elements of the Web-based program were the drivers of the behavior change. However, the enhanced program version achieved a greater retention rate, which in the longer term is critical for maximizing program reach and opportunity to facilitate maintenance of lost weight. There were advantages for those in the enhanced program group related to the extent of improvements in several secondary outcomes compared with the controls, including decreases in waist circumference, plasma total cholesterol, blood pressure, energy intake, and steps per day. Overall, both program versions provided important reductions in several risk factors for cardiovascular disease, as well as improvements in domains of quality of life and eating behavior.

In the only previous RCT of a commercial Web-based weight-loss program [11,12], the commercial Web-based program was found to be less effective than a self-help manual [11] and a more structured Web-based behavioral weight-loss program [12]. The eDiets commercial Web-based program had some similar program features to The Biggest Loser Club program, such as weekly self-monitoring of weight, meal and exercise plans, educational materials, and social support via a discussion board. Although eDiets also offered alternative sources of social support such as Web-based meetings and chat rooms, it did not provide a tool for participants to monitor and receive feedback on their dietary intake and physical activity, nor were participants reminded to use the program, which differs from the program in the current study. Therefore, we hypothesized that the mean weight losses for the basic and enhanced versions after 12 weeks in this study would be greater than those from the 2004 RCT using eDiets (–0.9% after 16 weeks) [11]. Interestingly, the 12-week weight loss in the current study is similar to the results of a more recent RCT using eDiets, which reported a mean weight loss of –3.6% after 6 months [12], which may indicate that developments in online capabilities facilitate program effectiveness.

This is the first RCT to examine the efficacy of a commercial program that specifically sought to include enhanced features (provision of additional personalized e-feedback, reminders, and phone calls) compared with the basic commercial program version without these features. We have shown that participants using the enhanced program did not lose significantly more weight after 12 weeks than did those in the basic program. This finding is not consistent with previous RCTs comparing basic versus enhanced versions of Web-based programs, as these have typically demonstrated greater weight loss in the enhanced study arm [12,23-27]. However, the basic programs in these previous studies had fewer program features and typically did not include all of the key components of Web-based interventions reported to be essential, such as being based on a theoretical framework, providing diet and physical activity feedback to participants, and having interactive features [28,29]. For example, Rothert et al’s basic Web-based program provided information only [27], while the enhanced features in other studies commonly used more human interventions, such as individualized human e-feedback generated by a therapist [24-26] or Web-based chat sessions conducted by a trained therapist [12]. The lack of difference between the basic and enhanced program in the current study, along with the magnitude of the weight lost after 12 weeks, suggests that either format of the program can produce clinically important initial weight loss. However, it also suggests that the enhanced program may require a greater number or intensity of enhanced features to be more effective than the basic version, or that the enhanced features provided are not necessary for many participants.

Although no significant difference in weight loss between basic and enhanced groups was observed, the enhanced group achieved significantly greater improvements in waist circumference than the basic group. They also demonstrated greater improvements in blood pressure, plasma total cholesterol, steps per day, measures of general health, and reduction in total daily energy intake than the control group, whereas the basic group did not. This suggests that the enhanced program offers additional benefit to participants in terms of risk factor reduction and in achieving behavior change that may assist with long-term maintenance of lost weight. Furthermore, the basic group were more likely to drop out of the study (25%) than the enhanced group (15%). The impact of this should not be overlooked because weight-loss success is associated with greater adherence to the prescribed treatment plan, and retention within treatment is the primary component of program adherence. Further follow-up of these participants will determine whether greater initial improvements in indicators of health status, quality of life, dietary intake, and physical activity, as well as higher initial retention, affect weight-loss outcomes in the long term and whether improvements are sustained over time. This will be important in evaluating the long-term efficacy of the basic program compared with the enhanced program.

This is one of the first RCTs of a Web-based weight-loss program to comprehensively assess secondary outcomes and to capture the potential of the program to have significant health benefits, irrespective of the weight loss achieved. To our knowledge, no other RCT evaluating Web-based weight-loss studies have assessed changes in lipids or insulin levels, and only one has evaluated changes in glucose levels [26]. The current study also demonstrated significantly greater reductions in blood pressure (systolic and diastolic) among the enhanced program users. Only two other RCTs evaluating Web-based weight-loss programs have assessed changes in blood pressure [30-32], with one of these finding greater reductions in systolic blood pressure among male participants only. Also, these improvements were demonstrated after 12 months [31], but not after 3 or 6 months [32]. Therefore, this is the first Web-based weight-loss RCT to demonstrate significant improvements in plasma total cholesterol and blood pressure after 12 weeks via participation in a Web-based weight-loss program that provides comprehensive personalized feedback and reminders to engage with the program.

Only a few Web-based weight-loss RCTs have reported changes in dietary intake and physical activity, and assessed difference in change across study arms [23-26,32-35]. To date, no study has demonstrated significant differences in dietary intake or physical activity change among participants of Web-based weight-loss programs compared with control groups, other treatment formats (eg, face-to-face), or enhanced Web-based programs. The current study found significantly greater reductions in mean total daily energy intake and increases in steps per day in the enhanced program users, which suggests that additional personalized feedback and reminders to use the program had positive influences on food and physical activity behaviors. However, further detailed investigation is needed to examine how participants change their food and physical activity behaviors in response to Web-based intervention. Further, no previous Web-based weight-loss studies have measured components of appetite and hunger using the TFEQ-R18. We have demonstrated improvements in both intervention groups compared with a control group in the domains of cognitive restraint and uncontrolled eating, but no significant improvements in emotional eating scores. Previous research has highlighted the association between appropriate eating patterns, such as higher dietary restraint and less emotional eating, and long-term weight-loss maintenance [36], and therefore this is an area where the current program could be refined. Longer-term follow-up of participants will demonstrate whether these initial improvements in eating patterns can be sustained or improved, and whether this affects weight-loss maintenance.

One other Web-based weight-loss study has measured quality of life. McConnon and colleagues found significant improvements in quality of life in Internet and usual-care groups with no significant difference between groups [33]. Therefore, the finding in the current study of significant improvements in quality of life, namely the general health domain of the SF-36, after 12 weeks’ participation in the enhanced version of the program is important.

A limitation of the current study is that all participants did receive human contact when they attended the clinical research center for clinical assessments. However, they were assessed by blinded assessors. Further, we gave them no advice on how to log in or engage with the program, other than giving them their login details. This was to simulate real-world engagement and use of the program, making the results generalizable to the overweight population of adults using commercial weight-loss programs. Due to the inclusion of a control group, the study also did not consider the potential differential impact of the Web-based programs as a result of participants’ varying levels of website usage. Study strengths include the use of a control group compared with two versions of the Web-based program, as well as the robust study design and the use of predominantly objective measures.

The results of this study demonstrate that Web-based weight loss can be efficacious across a range of weight-related outcomes and lifestyle behaviors, and that commercial providers can deliver effective Web-based programs targeting this important public health issue. Further study is needed to examine longer-term outcomes and whether Web-based programs with enhanced program features, including provision of personalized feedback, can retain people in the long term and lead to a greater treatment impact over time.

## Multimedia Appendix 1

CONSORT eHealth checklist V1.6 [37].

## Abbreviations

BMI: body mass index
HDL: high-density lipoprotein
LDL: low-density lipoprotein
MET: metabolic equivalent
RCT: randomized controlled trial
TFEQ: Three-Factor Eating Questionnaire-R18
